# Meniscofibular Ligament: Morphology and Functional Significance of a Relatively Unknown Anatomical Structure

**DOI:** 10.1155/2012/214784

**Published:** 2012-06-28

**Authors:** K. Natsis, G. Paraskevas, N. Anastasopoulos, T. Papamitsou, A. Sioga

**Affiliations:** ^1^Department of Anatomy, Medical School, Aristotle University of Thessaloniki, 54124 Thessaloniki, Greece; ^2^Department of Histology-Embryology, Aristotle University of Thessaloniki, 54124 Thessaloniki, Greece

## Abstract

*Purpose*. A relatively unknown ligamentous structure of the posterolateral corner of the knee joint, the so-called meniscofibular ligament (MFL), was investigated as regards its macroscopic morphology, its histological features, and its reaction to knee movements. *Material and Methods*. MFL was exposed on 21 fresh-frozen unpaired knee joints. Its microscopic morphology was examined utilizing for comparison the fibular collateral and the popliteofibular ligament. *Results*. MFL was encountered in 100% of the specimens as a thin striplike fibrous band extending between the lower border of the lateral meniscus and the head of the fibula. MFL was tense during knee extension and external rotation of the tibia, whereas its histological features were similar to those of fibular collateral and popliteofibular ligament. *Discussion*. Its precise histological nature is studied as well as its tension alterations during knee movements. The potential functional significance of the MFL with respect to its role in avoidance of lateral meniscus and lateral coronary ligament tears is discussed. *Conclusions*. MFL presumably provides an additional protection to the lateral meniscus during the last stages of knee extension, as well as to the lateral coronary ligament reducing the possibility of a potential rupture.

## 1. Introduction

The posterolateral corner of the knee joint is an anatomical area where serious interest has been appeared recently by anatomists as well as orthopaedic surgeons. The complexity of the anatomical structures constituting this area and the confused nomenclature of the ligaments and capsular thickenings concentrate the interest of many researchers [1–4]. Such a raised interest is due to the fact that posterolateral corner injuries with or without cruciate ligaments ruptures can lead to an unexplained instability [3]. The posterolateral corner elements prevent varus angulation, posterior shift, and excessive external rotation of the knee [5].

As regards lateral meniscus connections with the surrounding tissues, special attention has been paid to the meniscofemoral ligaments, the coronary ligament, and the popliteomeniscal ligaments-fascicles [6–8]. However, little data exists with regards to a relatively unknown and difficultly identified capsular thickening of the posterolateral corner, the so-called meniscofibular ligament (MFL) [9, 10]. MFL in animals very early in 1942 was included in Haines' original drawings, without any relative mention within the manuscript of his work [11]. However, Zivanovic later in 1964 prescribed comprehensively the morphological features and the potential functional properties of the MFL in humans [12]. The purpose of the present study is to investigate the presence and microscopic morphology of MFL, its reaction to knee movements, and its histological features. Moreover, we discuss the ligaments anchored to the lateral meniscus, as well as the morphological features and potential MFL functions.

## 2. Material and Methods

The potential presence of the MFL at the area of the posterolateral corner of the knee joint was detected on 21 fresh-frozen unpaired knee joints utilized for educational and research purposes at the Laboratory of Anatomy of the Medical School of the Aristotle University of Thessaloniki. In specific and according to the procedure of classical method of anatomical practice, the popliteal fossa was opened by routine dissection. The superficial to the popliteus tendon anatomical structures were removed to provide a clear vision for observing the capsular and ligamentous connections of the lateral meniscus. The aforementioned connections were repeatedly recorded as photographs during the course of the dissection. Furthermore, we noticed the changes of MFL tension during knee flexion and extension. At last, we excised the MFL along with its attachments to the head of fibula and the lateral meniscus, and we conducted appropriate histological examination in order to determine the precise histological nature of MFL. For comparison we performed histological examination in sections taken by the adjacent fibular collateral and the popliteofibular ligament in order to determine more accurately the histological nature of the encountered meniscofibular band. The tissues were stained with hematoxylin and eosin. For better histological analysis of the MFL elastin staining was utilized.

## 3. Results

**Figure 1:**
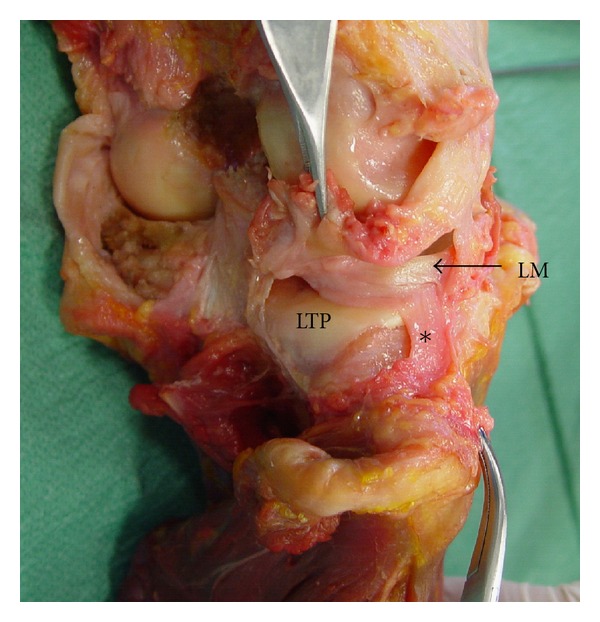
Posterolateral aspect of the right knee joint after excision of the overlying anatomical structures. The fibrous capsule and the lateral coronary ligament have been removed to provide a clear vision of the meniscofibular ligament (*) (LM: lateral meniscus, LTP: lateral tibial plateau).

**Figure 2:**
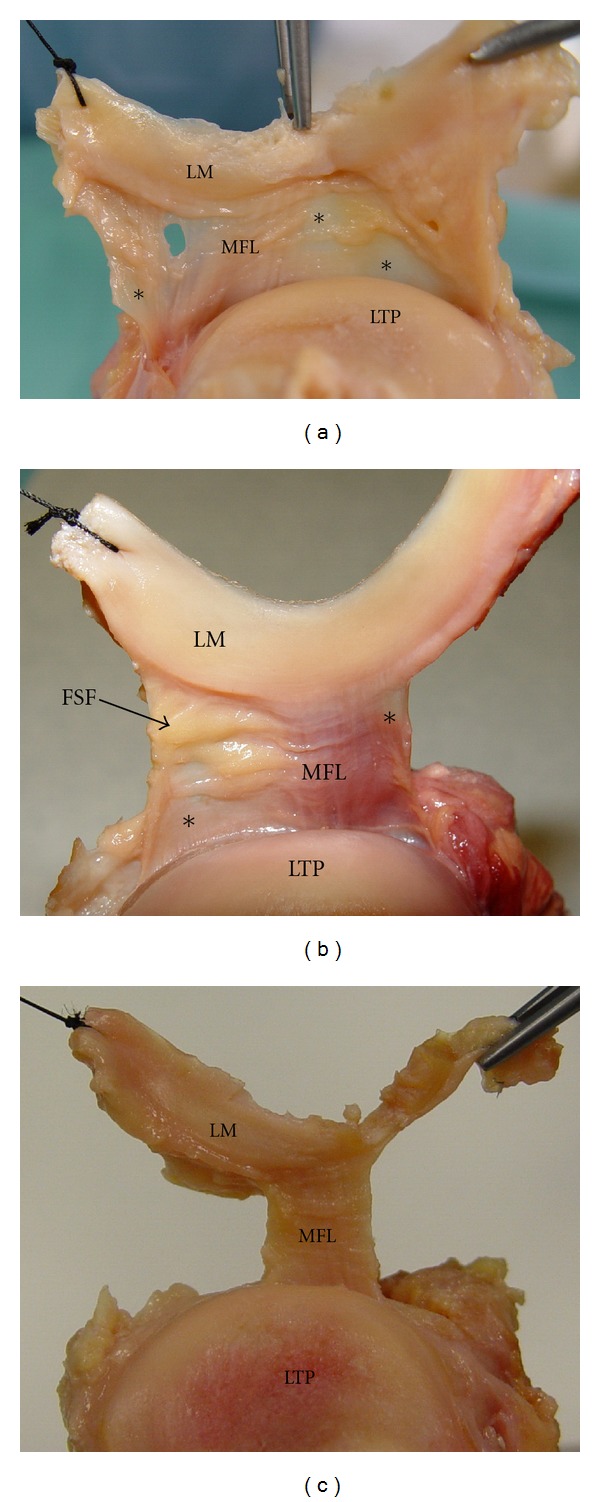
(a) The lateral meniscus (LM) has been raised and the lateral coronary ligament (*) along with the meniscofibular ligament (MFL) is demonstrated (LTP: lateral tibial plateau). (b) Fatty synovial folds (FSF) are shown covering part of the lateral coronary ligament. (c) The coronary ligament has been resected to allow better visualization of the MFL.

In 21 examined cadaveric unpaired knee joints (11 right-sided and 10 left-sided), thus an incidence of 100%, we noticed the existence of a thin fibrous band originating from the inferior border of the lateral meniscus at the area of the posterior part of its midportion. That band, the so-called MFL, directed backwards, outwards, and inferiorly anchored ultimately to the head of the ipsilateral fibula with the knee fibrous capsule attaching just proximal to the fibular head (Figure 1). MFL was seen to reinforce the thin lateral coronary ligament, which was extended from the lateral meniscus to the lateral aspect of the lateral tibial condyle just distal to the articular margin and proximal to the knee fibrous capsule attachment (Figure 2). In only one case (4.8%) MFL was hypoplastic, being, however, distinct. Performing knee movements we observed that MFL was tense during knee extension and external rotation of the tibia, whereas it was slack during the reverse movements.

**Figure 3:**
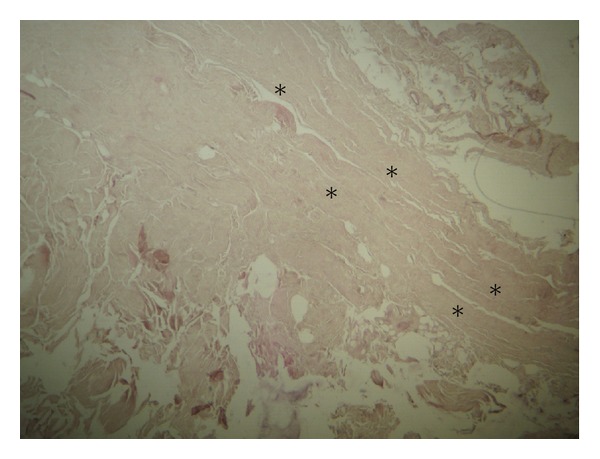
Demonstration of the histologic nature of the meniscofibular ligament (*) that consists of regular dense connective tissue with few extracellular matrices (hematoxylin and eosin staining, magnification ×16).

**Figure 4:**
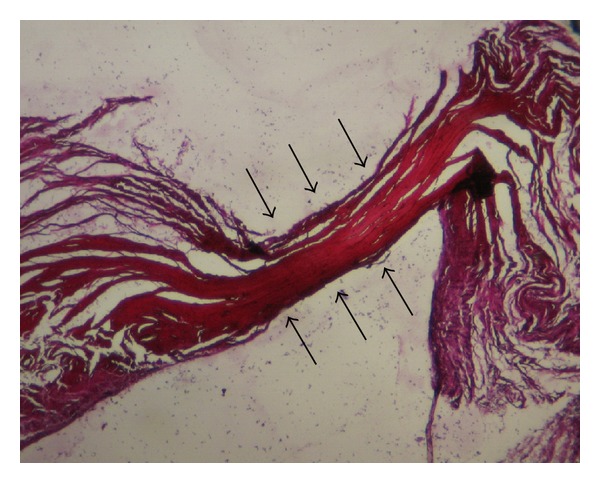
Numerous elastic fibres at the central area of the inferior portion of the meniscofibular ligament is shown (elastin staining, magnification ×16).

**Figure 5:**
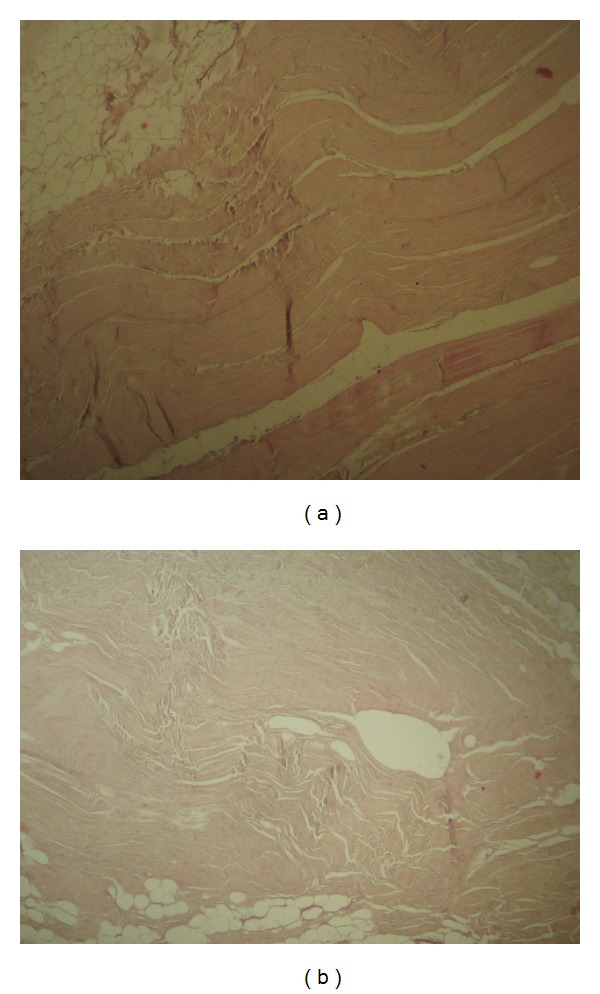
Histological structure of the adjacent fibular collateral ligament (a) and popliteofibular ligament (b) does not differ from that of the meniscofibular ligament (hematoxylin and eosin staining, magnification ×16).

The conducted histological examination demonstrated that MFL consisted of dense regular connective tissue with few extracellular matrices (Figure 3). At the central area of the inferior portion of the MFL plenty elastic fibers were detected (Figure 4). Furthermore, we concluded that no difference in microscopic morphology between MFL and fibular collateral and popliteofibular ligament was documented (Figure 5). It should be reported, however, that MFL is a ligament extending between meniscus and bone, such as meniscofemoral ligaments.

## 4. Discussion

The lateral meniscus, as it is well known, is less firmly anchored than the medial one as it is attached mainly to the fibrous capsule, via weak fibers [13]. However, lateral meniscus attachments to its neighboring structures do exist. In specific, the posterior horn is attached to the medial condyle of the femur anterior and posterior to the attachment to the posterior cruciate ligament, forming the anterior and posterior meniscofemoral ligaments [14–16]. At least one meniscofemoral ligament has been found in approximately 93% of knees, whereas 50% of them had both ligaments [17]. These ligaments can assist to withstand tibial posterior draw [18] and may act as a splint to keep the posterior cruciate ligament in position while it heals after rupture [8]. The anterior meniscofemoral ligament (of Humphrey) is slack in the extended knee and tightens with knee flexion to withstand tibial posterior draw. The converse occurs with the posterior meniscofemoral ligament of Wrisberg [8]. 

As regards the popliteus tendon, it provides almost consistently two fascicles, the superior, superomedial, or posterosuperior and the inferior, inferolateral, or anteroinferior bundle, which have been prescribed analytically firstly by Staubli and Birrer in 1990 [7, 19]. The presence of a third popliteomeniscal fascicle, the so-called posteroinferior fascicle, has been mentioned in the literature [6, 20]. The anteroinferior or inferolateral popliteomeniscal fascicle extends from the anterior margin of the popliteus tendon to the middle third of the lateral meniscus, whereas the posterosuperior or superomedial fascicle extends from the posterior margin of the tendon to the posterolateral aspect of the meniscus [21]. The aforementioned fascicles stabilize the lateral meniscus and when ruptured the mobility of the lateral meniscus is increased [19]. In addition, a portion of the joint capsule extending from the lateral edge of the lateral meniscus to the lateral tibial condyle, the so-called lateral coronary or meniscotibial ligament, secures the posterior horn of the lateral meniscus to the tibia [6, 22] and may be injured after excessive rotation of the knee [1]. Although the precise function of the coronary ligament has not been highly enlightened, it is supposed to play a role in keeping the lateral meniscus adherent to the lateral tibial plateau [23]. That ligament is responsible for limiting rotational movements of the knee, whereas it permits controlled anterior and posterior movement of the lateral meniscus [24]. Coronary ligament rupture may lead to increased mobility of the lateral meniscus, posterior knee pain, and a gap in the posterior capsule [25].

An additional ligament attached to the lateral meniscus, relatively unknown and neglected in the literature, is the meniscofibular ligament (MFL), a tapelike fibrous band extending between the inferior border of the lateral meniscus and the head of the fibula. That ligament was firstly described in 1964 by Zivanovic with an incidence of 78% of the 241 European knee joints examined [12]. Later, the same author observed that ligament in 80% of East African Bantu knee joints [9], whereas Bozkurt et al. observed the MFL in 100% of their specimens, attributing the lower incidence noticed in Zivanovic's study to the lack of detection of the specifically thin MFLs appeared in some materials [10]. Similarly MFL incidence was found to be 100% in our study, with only one case (4.8%) being hypoplastic but macroscopically apparent.

As regards the morphological features of the MFL, Zivanovic found the average width to be between 8 and 13 mm and the average length between 13 and 22 mm and while MFL was less than 1 mm thick [9]. The previously mentioned author claimed that MFL size was depending on the age and stature of the examined individual without providing further data. Bozkurt et al. found the mean thickness of the MFL to be 3.84 mm, ranging from 2.6 mm to 6.1 mm, including the capsule to which it adheres [10]. Such a thickness was found to be greater than the thickness detected by Zivanovic, a fact attributed to the capsule thickening that was coestimated in the performed measurements. In our research we observed that the MFL is incorporated into the lateral coronary ligament reinforcing its posterolateral segment. Bozkurt et al., however, detected the fibrous sheets located bilateral to the MFL as parts of the knee joint articular capsule, without defining them as lateral coronary ligament [10].

The MFL functional significance is a controversial issue. According to Zivanovic MFL has an opposite effect to that of the popliteal tendon limiting forward movement and medial gliding of the posterior horn of the lateral meniscus during the latter stages of knee extension, providing that way protection to the lateral meniscus [9]. Zivanovic found MFL as a permanent structure in the mountain gorilla and the cercopithecus, very well developed since in these animals MFL is more important for the protection of the lateral meniscus during knee extension [9]. MFL was loose in extreme flexion but it was very tense when the knee joint was extended. Zivanovic considered that during evolutionary development the appeared direct joint between the lateral femoral condyle and the head of fibula seen in the knees of tetrapods has disappeared, with MFL being a rudimentary structure [9]. The small MFL thickness noted in humans attributed to the assumption of the erect posture. 

On the contrary, Bozkurt et al. provided a potential relationship between MFL presence and the proximal tibiofibular joint [10]. They postulated that MFL may be responsible for backward and outward displacement of the lateral meniscus since the fibula rotates laterally during the dorsal flexion of ankle joint. It is worth mentioning that these authors noticed the MFL to be thicker in horizontal than in oblique proximal tibiofibular joints, where the fibula rotation is more restricted. It is supposed that in horizontal joints the MFL is loaded more than in oblique joints. Bozkurt et al. without providing the precise mechanism made an assumption that MFL has a protective effect in varus and external rotational traumas, whereas the same ligament may be presumably the cause of repetitive lateral meniscus tears [10]. 

In our study, where no evaluation of the MFL biomechanical properties was performed, we observed that the ligament was slack during knee flexion and medial rotation of the tibia and tense during knee extension and external rotation of the tibia, a notice that confirms Zivanovic's results. So, one hypothesizes that MFL could provide protection to the lateral meniscus from potential ruptures during the last stages of knee extension. In addition, we noticed that MFL reinforced the posterolateral portion of the lateral coronary ligament and not the posterior fibrous capsule of the joint, as noted by Bozkurt et al. [10]. Such an observation has not been mentioned previously in the literature for the best to our knowledge and we consider that such coronary ligament reinforcement provides protection to that ligament. Presumably, that fact supports and empowers the El-Khoury et al. notice that none lateral coronary ligament tear was encountered in excess of two thousands knee arthrograms [23]. The authors attributed this to the loose attachment of the lateral meniscus to the neighboring structures, the low incidence of ligamentous injuries over the lateral side of the knee, and the wide separation between lateral meniscus and collateral ligament. In addition, to the previous remarks we speculate that a further parameter leading to low frequence of lateral coronary ligament rupture is its potential reinforcement by the MFL. 

## 5. Conclusions

Analysing our observations one can speculate that MFL could offer protection to the lateral meniscus from likely damage during the last stages of knee extension. Moreover, MFL reinforced the posterolateral part of the lateral coronary ligament, a fact that could explain the relative low incidence of lateral coronary ligament rupture. Certainly, further investigation should be done to highlight the exact biomechanical characteristics of the MFL, as well as the likely relation or not to lateral meniscus tears, protection of the coronary ligament, and function and traumatology of the proximal tibiofibular joint.

## Abbreviations

MFL: Meniscofibular ligament.
